# Impact of stromal tumor-infiltrating lymphocytes (sTILs) on response to neoadjuvant chemotherapy in triple-negative early breast cancer in the WSG-ADAPT TN trial

**DOI:** 10.1186/s13058-022-01552-w

**Published:** 2022-09-02

**Authors:** Cornelia Kolberg-Liedtke, Friedrich Feuerhake, Madlen Garke, Matthias Christgen, Ronald Kates, Eva Maria Grischke, Helmut Forstbauer, Michael Braun, Mathias Warm, John Hackmann, Christoph Uleer, Bahriye Aktas, Claudia Schumacher, Sherko Kuemmel, Rachel Wuerstlein, Monika Graeser, Ulrike Nitz, Hans Kreipe, Oleg Gluz, Nadia Harbeck

**Affiliations:** 1 Department of Gynecology and Obstetrics, University Hospital Essen, Hufelandstrasse 55, 45147 Essen, Germany; 2 Institute of Pathology, Medical School Hannover, Hannover, Germany; 3 University Hospital Luebeck, Lübeck, Germany; 4 West German Study Group, Mönchengladbach, Germany; 5 Women’s Clinic, University Clinics Tuebingen, Tübingen, Germany; 6 Practice Network Troisdorf, Troisdorf, Germany; 7 Breast Center, Rotkreuz Clinics Munich, Munich, Germany; 8 Breast Center, City Hospital Holweide, Cologne, Germany; 9 Breast Center, Marien-Hospital, Witten, Germany; 10 Practice of Gynecology and Oncology, Hildesheim, Germany; 11 Department of Gynecology, University Hospital Leipzig, Leipzig, Germany; 12 Breast Center, St. Elisabeth Hospital, Cologne, Germany; 13 Breast Unit, Kliniken Essen-Mitte, Essen, Germany; 14 Department of Gynecology with Breast Center, Charité – Universitätsmedizin Berlin, Berlin, Germany; 15 Breast Center, LMU University Hospital, Munich, Germany; 16 University Hospital Hamburg-Eppendorf, Hamburg, Germany; 17 Breast Center Niederrhein, Ev. Hospital Bethesda, Mönchengladbach, Germany

**Keywords:** Triple-negative breast cancer, sTils, Neoadjuvant chemotherapy, Pathologic complete response, 3-Week biopsy

## Abstract

**Background:**

Higher density of stromal tumor-infiltrating lymphocytes (sTILs) at baseline has been associated with increased rates of pathological complete response (pCR) after neoadjuvant chemotherapy (NACT) in triple-negative breast cancer (TNBC). While evidence supports favorable association of pCR with survival in TNBC, an independent impact of sTILs (after adjustment for pCR) on survival is not yet established. Moreover, the impact of sTIL dynamics during NACT on pCR and survival in TNBC is unknown.

**Methods:**

The randomized WSG-ADAPT TN phase II trial compared efficacy of 12-week nab-paclitaxel with gemcitabine versus carboplatin. This preplanned translational analysis assessed impacts of sTIL measurements at baseline (sTIL-0) and after 3 weeks of chemotherapy (sTIL-3) on pCR and invasive disease-free survival (iDFS). Predictive performance of sTIL-0 and sTIL-3 for pCR was quantified by ROC analysis and logistic regression; Kaplan–Meier estimation and Cox regression (with mediation analysis) were used to determine their impact on iDFS.

**Results:**

For prediction of pCR, the AUC statistics for sTIL-0 and sTIL-3 were 0.60 and 0.63, respectively, in all patients; AUC for sTIL-3 was higher in NP/G. The positive predictive value (PPV) of “lymphocyte-predominant” status (sTIL-0 ≥ 60%) at baseline was 59.3%, though only 13.0% of patients had this status. To predict *non-pCR*, the cut point sTIL-0 ≤ 10% yielded PPV = 69.5% while addressing 33.8% of patients.

Higher sTIL levels (particularly at 3 weeks) were independently and favorably associated with better iDFS, even after adjusting for pCR. For example, the adjusted hazard ratio for 3-week sTILs ≥ 60% (vs. < 60%) was 0.48 [0.23–0.99]. Low cellularity in 3-week biopsies was the strongest individual predictor for pCR (in both therapy arms), but not for iDFS.

**Conclusion:**

The independent impact of sTILs on iDFS suggests that favorable immune response can influence key tumor biological processes for long-term survival. The results suggest that the reliability of pCR following neoadjuvant therapy as a surrogate for survival could vary among subgroups in TNBC defined by immune response or other factors. Dynamic measurements of sTILs under NACT could support immune response-guided patient selection for individualized therapy approaches for both very low levels (more effective therapies) and very high levels (de-escalation concepts).

*Trial registration*: Clinical trials No: NCT01815242, retrospectively registered January 25, 2013.

**Supplementary Information:**

The online version contains supplementary material available at 10.1186/s13058-022-01552-w.

## Background

Triple-negative breast cancer (TNBC) is defined by both absence of hormone receptor expression (i.e., estrogen receptor (ER) and progesterone receptor (PR)) and lack of overexpression/amplification of HER2/neu. It accounts for about 10 to 15% of cases [1]. Patients with early TNBC generally suffer from an unfavorable prognosis, particularly when they are diagnosed at young age [2]. However, among cases with response to neoadjuvant chemotherapy, prognosis may be more favorable [3, 4].

Chemotherapy is frequently administered in the neoadjuvant (preoperative) setting among patients with high-risk, early-stage breast cancer, including TNBC. Pathological complete response (pCR) is commonly defined by the absence of invasive tumor cells in the breast and axilla after neoadjuvant chemotherapy and represents optimal response to the regimen applied. It has been demonstrated among patients with high-risk, early breast cancer such as TNBC that pCR constitutes an early surrogate survival marker [5]. For this reason, trials testing efficacy of novel agents [6] are often designed with pCR as an endpoint. From a clinical perspective, however, identification and validation of even earlier biomarkers would provide substantial benefit, particularly in the context of window-of-opportunity study concepts [7].

The randomized prospective WSG-ADAPT TN phase II trial was designed to compare efficacy of carboplatin versus gemcitabine (each in combination with a nab-paclitaxel backbone) as a 12-week anthracycline-free chemotherapy regimen. pCR rates were higher in the carboplatin arm (45.9% vs. 28.7%, *p* = .002, OR = 2.11 [1.33–3.35]). The trial also required patients to undergo tumor biopsy after 3 weeks of therapy, which was subjected to translational analysis with the goal of identifying early biomarkers for response. For instance, one such surrogate biomarker for “early therapy response” consisted in a Ki-67 decrease > 30% or < 500 invasive tumor cells in the three-week serial biopsy (i.e., proliferation decrease after the first therapy cycle or early necrotic morphological changes, respectively). pCR rates were higher in “responders” than in “non-responders” (44.4% vs. 19.5% *p* < .001) [8].

Stromal tumor-infiltrating lymphocytes (sTILs) are associated with a patient’s immune response and offer a potentially favorable biological characteristic. Indeed, presence of sTILs is associated with increased pCR rates after neoadjuvant chemotherapy among all breast cancer subtypes [9]. According to pooled analysis of Denkert et al., a pCR advantage of higher TILs was evident among all subtypes including HR-positive breast cancer, but favorable survival impact was confirmed only in HER2-positive and TN breast cancer subtypes [10]. Most [11] but not all studies [12] reveal better prognosis for TNBC patients with high compared to low sTIL levels. In breast cancer, previous studies have identified a subtype referred to as *lymphocyte-predominant breast cancer* with a particularly high infiltrate at baseline ≥ 60% [10].

In the neoadjuvant context, favorable immune response could influence survival not only “indirectly” by more effectively obliterating residual primary tumor (i.e., mediation by pCR), but also “directly” via the subsequent long-term impact of immune processes. The data of this trial can provide evidence supporting either or both of these mechanisms, as we shall shortly see.

There is a limited body of evidence regarding the value of dynamic sTIL measurement during neoadjuvant chemotherapy. For instance, Luen et al. evaluated 375 samples retrieved from patients with TNBC who presented with residual disease (RD) following neoadjuvant chemotherapy. The authors observed a moderately strong positive correlation (*ρ* = .41) between sTIL levels in RD and density of T cell infiltrates. Furthermore, low sTIL levels were significantly associated with increased tumor size and more advanced nodal stage after neoadjuvant chemotherapy as well as inferior recurrence-free and overall survival [13]. A potential association was reported between sTIL measurements after 3 weeks of HER2-directed therapy and pCR in patients with early HER2-positive breast cancer [14]. However, the role of short-term (i.e., 3-week) sTIL dynamics during (specific) chemotherapy regimens among patients with TNBC remains uncertain.

The aims of the present translational analysis were (i) to quantify the dynamics of sTIL levels under neoadjuvant therapy in TNBC and their associations with clinical/pathological variables; (ii) to analyze the impact of TIL levels at baseline (sTIL-0) and at 3 weeks (sTIL-3) as well as of their dynamics on pCR; and (iii) to quantify the corresponding impacts on invasive disease-free survival (iDFS), taking into account pCR as an independent predictor of favorable survival and as a potential “mediator” for the impact of TILs.

## Methods

### Design of WSG-ADAPT TN

Design of the trial was described previously [8] (CONSORT: Additional file 1: Fig. S1). Patients with centrally confirmed early TNBC were eligible as follows: ER/PR expression < 1%; HER2 expression 0–1 or negative fluorescence in situ hybridization ratio < 2.0 if 2 + expression by immunohistochemistry; stages cT1c–cT4c, cN0/+. Inclusion criteria also specified: age > 18 years old, performance status of 0 or 1 and adequate organ function qualifying patients for chemotherapy. Enrolled patients were randomly assigned to one of the neoadjuvant trial arms in a 1:1 ratio:

- NP/G: nab-paclitaxel 125 mg/m^2^/gemcitabine 1000 mg/m^2^ d1,8,15 (q3w); versus
- NP/C: nab-paclitaxel 125 mg/m^2^/carboplatin AUC2 day 1,8 q3w.

Primary endpoint was pCR, defined as no invasive tumor in the breast and lymph nodes [15]. All cases of pCR and the majority of cases with non-pCR were assessed by surgery; however, in 30 patients non-pCR status was confirmed by core biopsy only (arms A/B: 15 (12%)/15 (19%)). Among those cases, further EC chemotherapy was given before surgery and four additional cases of pCR were reported.

The trial was powered for a pCR comparison by therapy arm and early response (defined as Ki-67 decrease > 30% or < 500 invasive tumor cells in the 3-week serial biopsy). A total of 336 patients were enrolled.

In patients without pCR at the time of planned surgery, additional anthracycline-containing chemotherapy (4 cycles epirubicin/cyclophosphamide) was mandatory; administration of this anthracycline-containing therapy as additional neoadjuvant chemotherapy (NACT) (after mandatory verification of the non-pCR status by core biopsy) or as additional adjuvant therapy was at investigator’s discretion. In patients with pCR, omission of additional chemotherapy (beyond the 12-week neoadjuvant study therapy) was permitted. Invasive disease-free survival (iDFS) was defined as survival without any invasive breast cancer relapse, second malignancy or death. The WSG-ADAPT TN trial was approved by the responsible ethics committees, federal authorities, and institutional review boards; it is registered with ClinicalTrials.gov (NCT01815242). All patients signed written informed consent.

### sTIL analysis

As part of a translational protocol, core-cut biopsies were obtained at baseline (as part of routine diagnostic work-up) as well as after 3 weeks of chemotherapy. In accordance with international guidelines, analysis was confined to quantification of stromal TILs (sTILs). Formalin-fixed and paraffin-embedded tissue was cut at 4–5 µm thickness and transferred to slides. Staining was performed using hematoxylin–eosin staining. Slides were digitalized using the Aperio ImageScope 12.0 software (Leica, Germany, version 12.3.0.5056) and analyzed both qualitatively and quantitatively at 20–40 × amplification. sTIL infiltrates in tumor-surrounding normal breast tissue as well as in DCIS were excluded, as were necrotic or fibrotic areas, areas of florid granulocytic inflammation and extensive regressive hyalinization, and areas of cell-free sclerosis. As previously recommended [13], sTIL counts were quantified as a percentage in relation to surrounding tumor tissue; levels ≥ 30% were (in all but one case) rounded to the nearest 10% (e.g., 30%, 40%, etc.), otherwise to the nearest 5% (e.g., 0%, 5%, etc.). Slides with < 500 cells available for analysis (generally due to tumor necrosis, lack of invasive tumor cells) were classified as “low cellularity.”

### Statistical analysis

This preplanned translational analysis focuses on the impact of sTILs among tumor samples at baseline (sTIL-0) and after 3 weeks of chemotherapy (sTIL-3) on pCR and iDFS in the ADAPT TN trial. ROC analysis was performed for both sTIL-0 and sTIL-3 to assess their overall predictive performance for pCR in terms of the AUC statistic and to evaluate the dependence of positive predictive value (PPV), sensitivity, and specificity on both sTIL-0 and sTIL-3 cutoffs. The analysis also provides the percentage above versus below each successive cutoff value.

Based on prior evidence from ring studies [16], the particular cutoff 60% was used to define a binary baseline sTIL status variable by coding “*TIL*+” for sTIL-0 ≥ 60% versus “*TIL−*” for sTIL-0 < 60%. For reasons explained in detail below, sTIL-3 data were coded in a nominal composite variable taking three possible (non-missing) values: *3wTIL*+ (sTIL-3 ≥ 60%), *3wTIL−* (sTIL-3 < 60%) and *3wLC* (low cellularity after three weeks). The six combinations of values of these two variables (e.g., TIL+ to 3wTIL−) were also coded as a nominal variable to define the combinatorically possible dynamic transitions. Differences of odds ratios (by treatment) were tested using normal approximations to the log-odds.

The Kaplan–Meier method was initially used to quantify the association of these markers with iDFS in all patients and in subgroups defined by pCR versus non-pCR; three-year iDFS (abbreviated 3y-iDFS), with 95% confidence intervals, was estimated for each subgroup. Subgroup comparisons were carried out using log-rank statistics; pairwise comparisons were computed in case of more than two subgroups.

In order to assess potential impacts of these markers on iDFS, it is highly informative to quantify the potential role of pCR as a statistical mediator for the impact of immune response on iDFS. To this end, adapting the classical methodology of Baron and Kenny [17] the odds ratios (OR) of the coded sTIL variables for pCR were first estimated by binary logistic regression. Next, for continuous coding and for each category of each sTIL variable (e.g., TIL+ vs. TIL−), a univariable and a multivariable Cox model for iDFS were estimated to compute corresponding unadjusted and adjusted (for pCR) hazard ratios, respectively. Tests of interaction between the sTIL variables and pCR were carried out to assess potential mediation (or lack thereof), i.e., the degree to which sTILs represent independent survival predictors—beyond their influence through pCR.

Differences of means between independent groups were tested by T-statistics. Pearson correlations (denoted *r*) were also assessed. Associations among categorical variables were assessed by chi-squared statistics or Fisher’s exact test. Quantities in square brackets indicate 95% confidence intervals throughout the results.

## Results

### Association of baseline sTILs with clinical/pathological parameters

Baseline sTIL (sTIL-0) measurements were available in 323 (96.1%) patients (mean 29.5%, SD 24.4%, median 20%). Associations between sTIL-0 measurements and clinical/pathological categories are summarized in Table 1. Node-positive status and central grade 3 were associated with higher sTIL-0 levels, while age and tumor size showed no significant association.

**Table 1:** Associations of population with available sTIL-0 values (*n* = 323) with clinical/pathological characteristics

Characteristic	Category	*n*	Mean sTIL-0 (%)	*p* value
Age at diagnosis*	≤ 50 yrs > 50 yrs	162 161	30.0 28.9	.68
Tumor stage	pT1 pT2-4	121 202	26.5 22.9	.10
Nodal status	Negative Positive	238 85	27.9 33.9	.049
Central grade	G 2 G 3	20 302	17.3 30.3	.021

*Median patient age at diagnosis was 50.5 yrs. (minimum 26 yrs., maximum 76 yrs.)

### Discrete coding of baseline and 3-weeek sTILs

As explained above, considering prior evidence, sTIL-0 was also coded as binary variable “baseline sTIL status”: “TIL+” (sTIL-0 ≥ 60%, known as “lymphocyte -predominant breast cancer”) versus “TIL−” (sTIL-0 < 60%). The same cutoff was used for sTIL-3, but a coding scheme for an attribution of missingness was required: Among the 110 samples with missing sTIL-3 measurements, 63 cases (57.3%) were attributable to “low cellularity.” For clinical interpretation, it is important to distinguish between missing sTIL-3 due to low-cellularity status and missingness for other reasons: Low cellularity is characterized by tumor necrosis and consequent lack of invasive tumor cells and is thought to result from extensive response to neoadjuvant therapy after 3 weeks. Since low-cellularity status represents a biological feature of response to therapy, it is likely to be *informative* for pCR and survival, whereas missingness for other reasons is presumably unrelated to therapy and less likely to be informative for pCR and survival. Therefore, a 3-week sTIL status variable was defined with three categories: In addition to the categories “3wTIL+” (TIL-3 ≥ 60%) and “3wTIL−” (TIL-3 < 60%), a third category “3wLC” was coded for “low cellularity” in the 3-week biopsy. With this coding, *n* = 286 patients were available for analysis.

### Dynamics of sTILs under NACT

sTIL measurements at 3 weeks (sTIL-3) were available in 226 (67.3%) patients (mean 38.4, SD 27.9%, median 30%). Paired sTIL-0 and sTIL-3 measurements were available in all 226 of these patients. The estimated Pearson correlation between sTIL-3 and sTIL-0 in paired measurements was *r* = 0.655 [0.572–0.723] (*p* < .001). The mean increase was 9.20% [6.32–12.09%] (*p* < .001, paired t-test).

Table 2 summarizes the transitions in the sTIL status variables from baseline to week 3 (including low cellularity) under NACT according to trial arm. In the trial as a whole, lymphocyte-predominant status (TIL+ vs. TIL−) at baseline was not predictive for low cellularity at 3 weeks: The percentages were 19.6% (TIL +) versus 21.2 (TIL−) (*p* = .79), suggesting that the estimated mean increase in sTIL levels on therapy (see above) could be relatively unbiased, despite the substantial proportion of missing values. The percentage of low-cellularity cases among patients with TIL+ at baseline was higher in the NP/C arm (*p* = .008), whereas the percentages among patients with TIL− at baseline were about the same. Evidently, after 3 weeks of therapy, the initial pool of patients with favorable sTIL levels at baseline appears to have been more strongly “depleted” (due to low cellularity) in NP/C than in NP/G.

**Table 2:** Dynamics of sTILs from baseline to 3 weeks by arm (*n* = 282)

Dynamics	3wTIL− *n* (%)	3wTIL+ *n* (%)	3wLC *n* (%)
*NP/G*			
TIL−	70 (53.4%)	36 (27.5%)	25 (19.1%)
TIL+	7 (25.0%)	19 (67.9%)	2 (7.1%)
*NP/C*			
TIL−	69 (69.7%)	10 (10.1%)	20 (20.2%)
TIL+	3 (12.5%)	12 (50.0%)	9 (37.5%)
*All patients*			
TIL−	139 (60.4%)	46 (20.0%)	45 (19.6%)
TIL+	10 (19.2%)	31 (59.6%)	11 (21.2%)

### Associations of sTIL-0 and sTIL-3 with pCR

For analysis of the association of sTIL-0 and sTIL-3 measurements with pCR, 311 and 223 patients were available, respectively. In patients with pCR (*n* = 110), mean sTIL-0 levels (36.0%) were more than 10% higher than in non-pCR patients (*n* = 201, mean = 25.7%) (*p* < .001). Similarly, sTIL-3 levels were more than 13% higher in patients with pCR (*n* = 63, mean = 47.9%) than in non-pCR patients (*n* = 160, mean = 34.8%) (*p* = .002). There was no significant association of changes in sTIL levels with pCR. The dynamics of sTILs and their association with pCR are illustrated in Additional file 2: Fig. S2 as a scatter plot of 3-week versus baseline measurements, marked by pCR status.

The significant association between sTIL-0 and pCR persisted when analyzed separately by treatment arm. In NP/G, mean sTIL-0 values of 40.1% versus 28.1% were observed among patients with (*n* = 49) versus without (*n* = 124) pCR, respectively (*p* = .004). In NP/C, mean sTIL values of 32.7% versus 21.5% were observed among patients with (*n* = 61) versus without (*n* = 77) pCR, respectively (*p* = .008).

Regarding 3-week levels (sTIL-3), the trial arms showed rather different behavior: In NP/G, sTIL-3 levels were more than 20% higher on average among patients with pCR (*n* = 29, mean = 58.7%) than among those without pCR (*n* = 102, mean 37.6%) (*p* < .001), whereas in NP/C, the difference was not significant (*p* = .12); mean sTIL-3 among pCR patients (*n* = 34) was 38.8% compared to 29.9% in non-pCR patients (*n* = 58).

Among patients with pCR, the percentage of those with 3wTIL+ was 35.3% in NP/G versus 17.9% in NP/C (*p* = .03). Among patients with non-pCR, the percentage of those with 3wTIL+ was 29.1% in NP/G versus 12.7% in NP/C (*p* = .006).

ROC analysis was carried out in order to explore the overall performance of both sTIL measurements as predictors of pCR versus non-pCR (Additional file 3: Fig. S3A/B). In particular, the AUC for sTIL-0 is 0.600 [0.531–0.668] (*p* = .004), while the AUC for sTIL-3 is 0.628 [0.545-0.712] (*p* = .003). For both measurements, overall performance is thus significantly, but only moderately better than random chance (AUC = 0.5).

Analyzing separately by trial arm, we find that the performance of sTIL-0 is comparable in NP/G (AUC = 0.609 [0.509-0.710], *p* = .025) and NP/C (AUC = 0.617 [0.521–0.713], *p* = .018), respectively. However, the performance of sTIL-3 in terms of AUC appears to be far greater in the NP/G arm (AUC = 0.711 [0.605-0.816], *p* = .001) than in the NP/C arm (AUC = 0.584 [0.460–0.709], *p* = .178), where it is not even significantly higher than random chance.

**Fig. 1:**
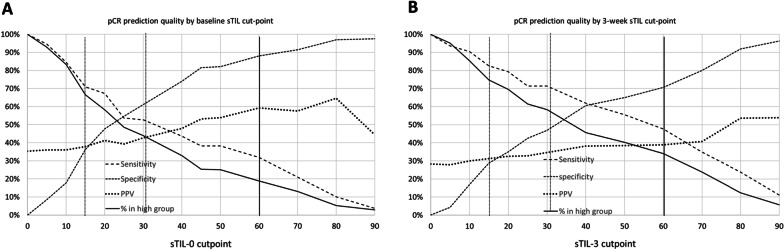
Sensitivity (long-dashed curves), specificity (short-dashed curves), PPV (dotted curves) and % addressed (solid curve) as a function of cut point in ROC analysis for **A** sTIL-0 and **B** sTIL-3 (lower panel). Vertical lines indicate particular cut points discussed in the text. The rightmost cut point corresponds to “lymphocyte-predominant” status

Figure 1A, B shows the variation of sensitivity, specificity, and positive predictive value as a function of cut point as well as the percentage of patients classified as “high” (≥ cut point) for sTIL-0 and sTIL-3, respectively. Since a considerable density of data is presented in these figures, we highlight the key performance statistics for sTIL-0 (obtainable from the graph) at selected cut points proposed in the literature. For the cutoff sTIL-0 ≥ 60% (lymphocyte -predominant breast cancer), 13.0% of patients had sTIL-0 above the cutoff (solid curve). Among those patients, the PPV (here, estimated conditional probability of having a pCR, given sTIL-0 ≥ 60%) was 59.3% (dotted curve); the specificity was 88.1% (short-dashed curve); however, the sensitivity was only 31.8% (wide-dashed curve), reflecting the low percentage in the high group defined by this cut point. For the cutoff sTIL-0 ≥ 30% used by Loi et al. [11], 44.0% are addressed (high group). In this group, the PPV is 42.6%, i.e., the majority of patients with sTIL-0 above the cut point still did not have pCR. The sensitivity was 52.7%, and the specificity was 61.2%. Finally, defining a cutoff as < 15% would put 33.8% in the “low” group. Here, it makes sense to discuss the prediction of non-pCR: The predictive value (probability of non-pCR, given sTIL-0 ≤ 10) was 69.5%, while the sensitivity was 36.3%, and the specificity was 70.9%. The performance of sTIL-3 at these or other cut points can be derived analogously from Fig. 1B.

For comparison with previous work, we note that the odds ratio associated with each 10% increase in sTIL-0 was 1.19 [1.08–1.31] (*p* < .001); the odds ratio associated with each 10% increase in sTIL-3 (among those with measurements) was 1.18 [1.06–1.31] (*p* = .002).

### Discrete variables and pCR

For both sTIL-0 and sTIL-3, the defined categories were strongly associated with pCR (Table 3, upper and lower panel, respectively). The odds ratio for TIL+ versus TIL− was 4.56 in NP/G and 2.59 in NP/C, but this apparently differing impact by trial arm was not significant (*p* = .35). The category 3wLC appears relatively favorable for pCR even in comparison with 3wTIL+ (*p* = .07, Hosmer–Lemeshow test). As we will shortly see, this relative favorability does not persist, however, with respect to iDFS:

**Table 3:** Associations between pCR and both sTIL-0 and sTIL-3 (upper panel: pCR by baseline sTIL categories; lower panel: pCR by 3-week categories)

Arm	sTIL-0 category	pCR %	OR	*p*	*n*
NP/G					173
	TIL−	22.0	1.00*		141
	TIL+	56.3	4.56 [2.04–10.20]	< .001	32
NP/C					138
	TIL−	39.6	1.00*		111
	TIL+	63.0	2.59 [1.09–6.17]	.032	27
Trial					311
	TIL−	29.8	1.00*		252
	TIL+	59.3	3.44 [1.92–6.18]	< .001	59
NP/G					160
	3wTIL−	14.5	1.00*		76
	3wTIL+	32.7	2.87 [1.23–6.74]	< .001	55
	3wLC	51.7	6.33 [2.40–16.68]	.032	29
NP/C					126
	3wTIL−	31.4	1.00*		70
	3wTIL+	54.5	2.62 [0.98–6.97]	.054	22
	3wLC	64.7	4.00 [1.68–9.51]	.002	34
Trial					286
	3wTIL−	22.6	1.00*		146
	3wTIL+	39.0	2.19 [1.20–3.98]	.011	77
	3wLC	58.7	4.87 [2.58–9.19]	< .001	63

*Reference category

### Association between sTIL-0 and sTIL-3 and IDFS

Median follow-up among surviving patients was 36 months. At this follow-up, the substantial advantage of the NP/C arm regarding pCR (OR = 2.11) was not reflected in a significant iDFS advantage. However, as previously reported [11], higher baseline sTIL levels were favorably associated with iDFS in univariate Cox analysis.

**Fig. 2:**
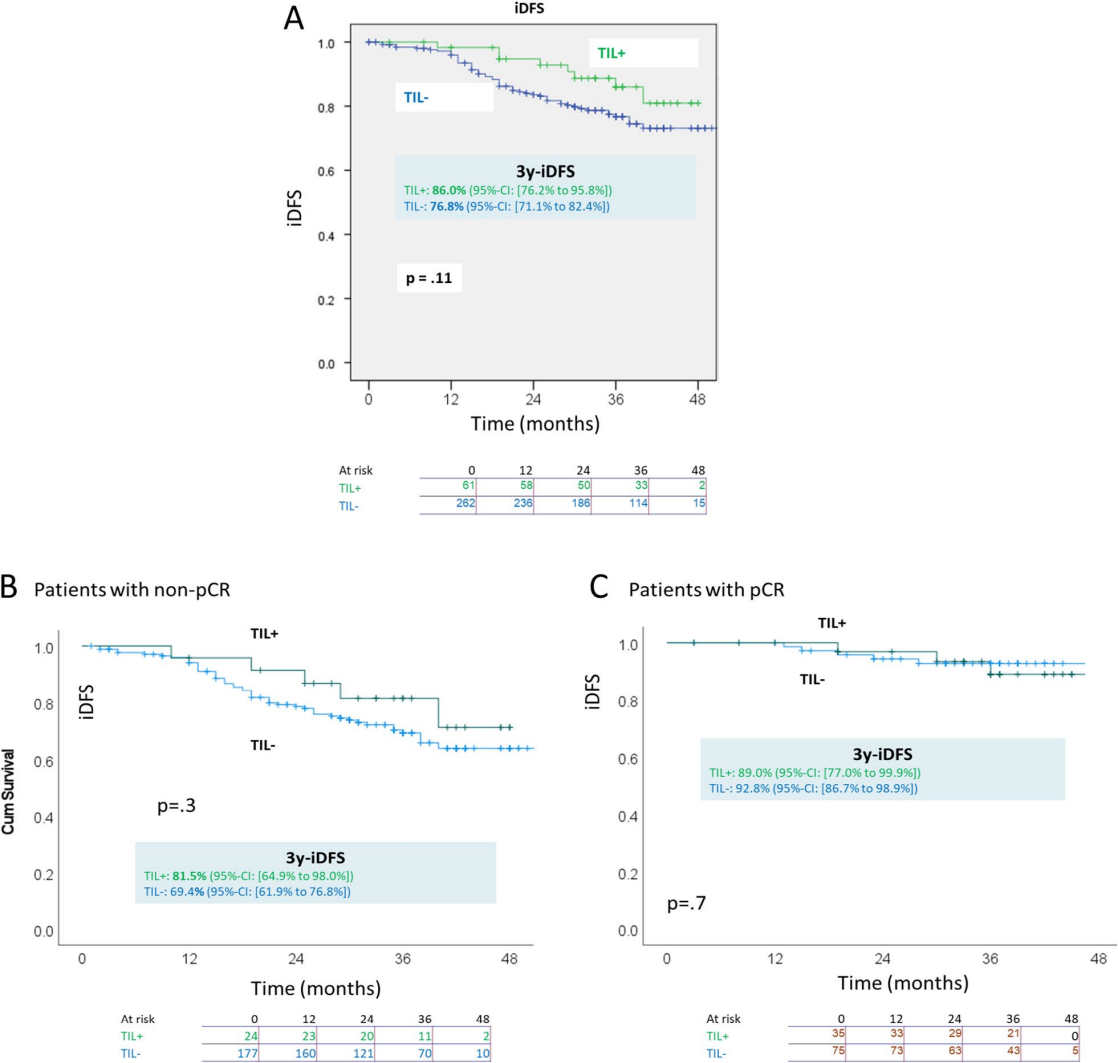
iDFS in Kaplan–Meier analysis for TIL+ (baseline lymphocyte-predominant status) versus TIL− in **A** all patients, **B** patients with non-pCR and **C** patients with pCR

Defining groups by dichotomized baseline sTILs: in all patients, the lymphocyte-predominant group (TIL +) had estimated 3y-iDFS of 86.0% (95%-CI [76.2% to 95.8%]), while the group with TIL− had 3y-iDFS of 76.8% (95%-CI [71.1% to 82.4%]) (*p* = .11, Kaplan–Meier, Fig. 2A). The corresponding iDFS curves in the subset of patients with non-pCR (residual disease) and in those with pCR are shown in Fig. 2B, C, respectively. Despite the visual impression of superior iDFS for TIL+ overall and in the non-pCR subset, the differences were not significant (recalling that follow-up was 36 months).

**Fig. 3:**
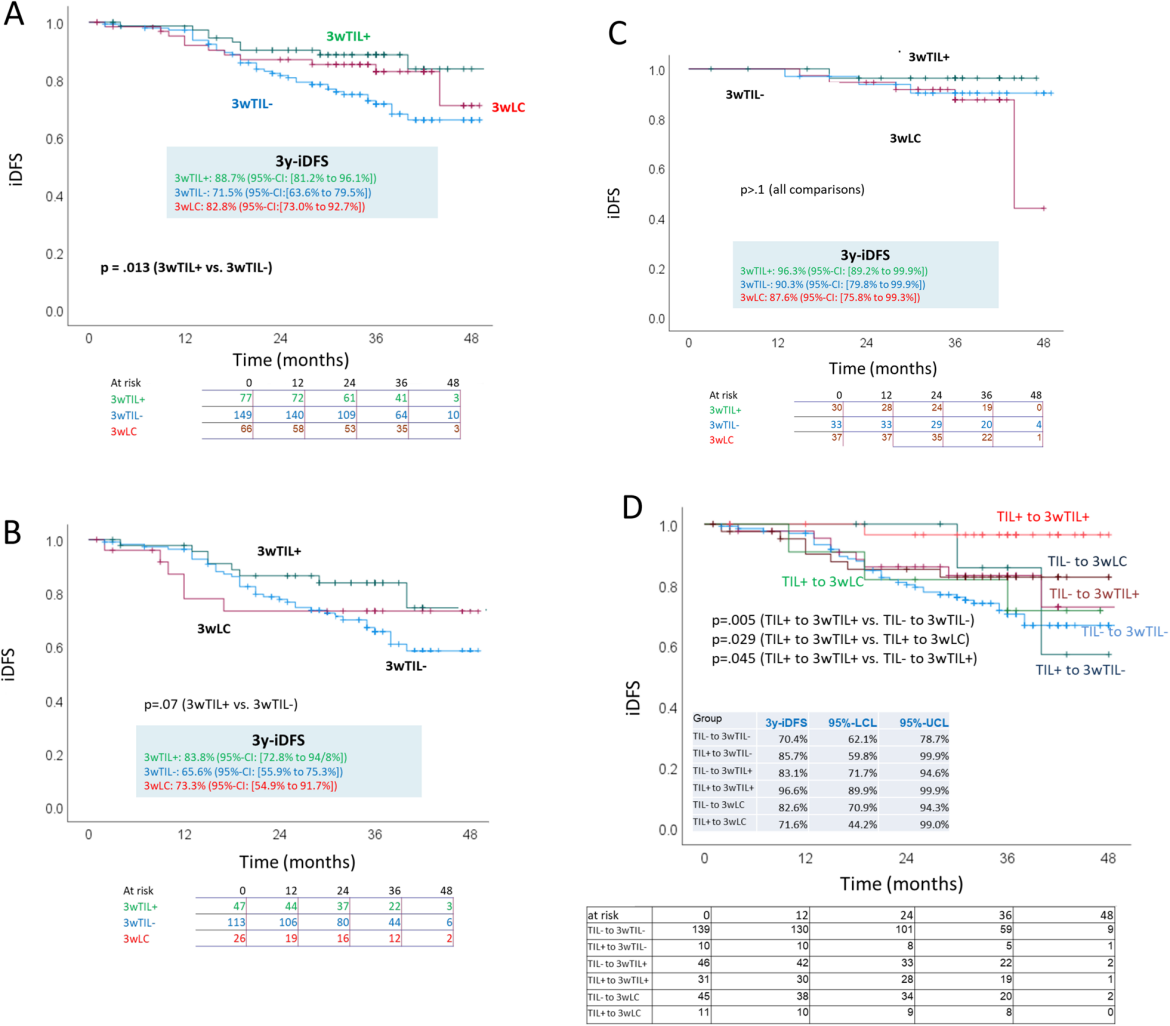
iDFS in Kaplan–Meier analysis for 3-week measurements, i.e., 3wTIL+ versus 3wTIL− versus 3wLC (low cellularity), in **A** all patients, **B** patients with non-pCR and **C** patients with pCR and **D** iDFS in Kaplan–Meier analysis for subgroups defined (see text) by sTIL transitions from baseline to 3 weeks

Regarding sTIL-3 (Fig. 3A): Defining groups according to the nominal composite variable coding described above, one finds a significant advantage for 3wTIL+ versus 3wTIL− in all patients, with about 17% higher 3y-iDFS; the group coded 3wLC (low cellularity) had iDFS between the other two groups). This advantage remains significant and similar in magnitude in the non-pCR subset as well (Fig. 3B) but not in the pCR subset (Fig. 3C).

The six combinatorically possible dynamic transitions of baseline to 3-week sTIL categories were coded as explained above in a nominal variable and analyzed for iDFS. The curves 3y-iDFS statistics and significant pairwise comparisons are shown in Fig. 3D. The most favorable combination was the transition TIL+ to 3wTIL+ with estimated 3y-iDFS of 96.6%. The superiority compared to the transition TIL− to 3wTIL− is unsurprising in view of the immediately preceding iDFS comparisons. However—recalling that for pcR, the 3wLC category was consistently more favorable than 3wTIL+ (Table 3)—for iDFS, the transition TIL+ to 3wTIL+ was far superior (25% higher 3y-iDFS) to the transition TIL+ to 3wLC. Despite low absolute numbers, this difference suggests that the reliability of pCR following neoadjuvant therapy as a surrogate for survival could vary among subgroups in TNBC. There was no significant association of the change in sTIL levels with iDFS.

### Mediation analysis

As explained above, mediation analysis according to the methodology of Baron and Kenny has performed to quantify the degree to which sTILs represent independent predictors of iDFS in this trial—beyond their influence through pCR. Mediation analysis can reveal the relative importance of distinct biological mechanisms for impact of immune response on survival under NACT in TNBC.

The first step of mediation analysis, demonstration of an impact of sTILs on pCR (favorable, e.g., OR 3.44 for TIL+ versus TIL−, OR 2.19 for 3wTIL+ versus 3wTIL−), was presented above for both baseline and 3-week measurements (Table 3). The next step is to show that pCR has a significant univariable impact on iDFS (HR = 0.27 [0.14–0.53] (*p* < .001)). Note however that this association may or may not imply causation.

The remaining mediation analysis consists of *univariate* Cox regression of each TIL variable on iDFS, followed by *multiple* Cox regression including pCR (with an interaction test); the results are summarized in Additional file 4: Table S1 for baseline sTIL and 3-week sTIL as *continuous variables* and in Additional file 4: Table S2 for baseline sTIL and 3-week sTIL *categories*. One then compares the adjusted HRs (including pCR) of the sTIL markers (continuous as in Additional file 4: Table S1 or categorical as in Additional file 4: Table S2) with their corresponding unadjusted HRs. Mediation is suggested if the adjusted and unadjusted HRs differ substantially.

We consider first the continuous models (Additional file 4: Table S1): For both sTIL-0 (baseline) and sTIL-3, there is no evidence for a substantial difference between adjusted and unadjusted HR (noting that sTIL-0 is not significant when pCR is included). We therefore find no evidence for mediation in this continuous analysis.

The analysis of sTIL categories (Additional file 4: Table S2) is slightly more complicated but offers the advantage that low cellularity is included: In the case of sTIL-0 (baseline), we see that while the uHR of 0.56 would suggest a stronger impact on iDFS than the adjusted HR of 0.77 for TIL+ versus TIL−, the corresponding HRs are not significant in either model, consistent with the log-rank tests of Fig. 1A–C. Had the uHR been significant, the difference would have suggested that a favorable effect of high baseline sTILs was at least partially mediated by pCR.

For the factor low cellularity at 3 weeks (3wLC), which was a stronger predictor of pCR than 3wTIL+, neither the unadjusted nor the adjusted HR was significant for iDFS. Again, any impact of low cellularity on iDFS was apparently either confounded with pCR or mediated by pCR. It is worth noting that the HR for pCR remained about the same in unadjusted analysis (see above) and in all models including TIL measurements (Additional file 4: Tables S1, S2).

What we see in the 3-week analysis for 3wTIL+ versus 3wTIL− is that the unadjusted hazard ratio estimate uHR = 0.42 was (only) slightly more favorable than the adjusted estimate aHR = 0.48, while both were significant. The lack of a significant difference was verified by interaction analysis. Hence, the data suggest that 3wTIL+ is a significant prognostic factor for iDFS, independent of pCR, whose impact is at most only partially mediated by pCR.

## Discussion

### Impacts of TILs on pCR and iDFS in the neoadjuvant TNBC context

This paper presents (to our knowledge) the first retrospective dynamic sTIL analysis based on patient samples acquired both at baseline and at 3 weeks within a prospective randomized clinical (de-escalated, 12-week) NACT trial in early TNBC, i.e., the ADAPT TN trial. In the present analysis, we have demonstrated favorable impacts of higher sTIL levels (measured either at baseline or at 3 weeks), not only on pCR, but also on invasive disease-free survival (iDFS). The favorable impact of higher sTIL levels (particularly when measured at 3 weeks) on survival was quantitatively independent of pCR, i.e., it was not simply mediated by pCR.

The independent impact of sTILs provides important clues to underlying tumor biological processes. Efficacy in obliterating primary tumor cells (reflected in pCR) does not necessarily imply efficacy for retarding subsequent proliferation and metastasis (reflected in iDFS), particularly if metastatic cells have a more aggressive clonality. For example, the clear advantage of NP/C over NP/G regarding pCR was not seen regarding iDFS. Moreover, low cellularity at 3 weeks was a strong predictor of pCR, but not for iDFS. In contrast, the above mediation analysis suggests that favorable immune response (particularly as quantified by the 3-week sTIL measurement) can influence key tumor biological processes such as proliferation and metastasis for long-term survival. The results imply that the reliability of pCR following neoadjuvant therapy as a surrogate for survival could vary among subgroups in TNBC defined by immune response or other factors.

While sTIL levels at the two measurement times were rather strongly correlated (*r* = 0.66), mean sTIL levels increased under NACT by nearly 10% among paired measurements. Response to NACT also included a substantial proportion of patients with “low cellularity” at 3 weeks, which almost certainly represents a favorable biological feature, but precluded measurement of sTILS. Baseline “lymphocyte-predominant” status (sTIL-0 ≥ 60%) was not predictive for low cellularity, suggesting that the observed mean increase in sTIL levels was not simply a selection effect. We did see evidence that after 3 weeks of NACT, the initial pool of patients with favorable sTIL levels at baseline was more strongly “depleted” (due to low cellularity) in NP/C than in NP/G, consistent with the fact that NP/C is also a more efficacious therapy regarding pCR.

ROC analysis showed that sTILS generally provide moderate performance for predicting pCR throughout the range of measurement at both time points, with the 3-week performance slightly better. While the predictive performance of sTIL-0 was comparable in NP/G and NP/C, the good performance of sTIL-3 was mostly attributable to data in the NP/G arm; performance was not significantly better than random chance in the NP/C arm. This observation is consistent with the aforementioned evidence of “depletion” of patients with favorable sTILs in NP/C but less so in NP/G.

Although much of the analysis here utilized the consensus [18] “lymphocyte-predominant” cut point defined by sTILS ≥ 60%, the evidence from our trial provided by ROC analysis does not strongly favor a particular sTIL cut point (at either measurement time) to answer all questions regarding prediction of pCR or iDFS. Use of continuous values has considerable merit, particularly regarding baseline measurements. Each of the cut points analyzed in detail here (15%, 30%, 60%) could offer advantages or disadvantages depending on clinical context. For example, lymphocyte-predominant status at baseline had high positive predictive value for pCR prediction but captured only 13% of patients, thus with low sensitivity. The 30% cutoff used by Loi et al. [11] does select more patients (44%), but only a minority actually had pCR. On the other hand, the 15% sTIL-0 cut point (sTIL ≤ 10% in our data) could be quite useful if the goal is to select patients with a high probability of *non-pCR;* it selected about 1/3 of patients, with almost 70% positive predictive value for non-pCR. We suggest that future trials or guidelines could benefit from an application-oriented approach based on ROC analysis rather than ad hoc specification of one universal sTIL cut point.

To our knowledge, ours is the first analysis of 3-week sTIL measurements during neoadjuvant therapy among patients with TNBC. However, Bai et al. analyzed the prognostic value of sTILs among tumor samples taken from TNBC patients with residual disease after neoadjuvant chemotherapy. The authors observed significantly better outcomes among patients with high CD4-TILs (DFS and OS) and high CD8-TILs as well as low CD20-TILs and suggested that TILs (or their subtypes) in residual disease might be used as a potential predictive biomarker of survival in TNBC patients after neoadjuvant chemotherapy [19].

In a pooled analysis of 2148 patients with TNBC from nine studies (all treated by different polychemotherapy regimens of 18–24 weeks of duration), Loi et al. demonstrated that increased presence of sTILs was significantly and independently associated with improvement in several prognostic end points including overall survival. In fact, each 10% increment in sTILs was shown to correspond to a hazard ratio of 0.87 (95% CI 0.83 to 0.91) for iDFS, 0.83 (95% CI 0.79 to 0.88) for DDFS, and 0.84 (95% CI 0.79 to 0.89) for OS [11]. Similarly, presence of sTILs has been associated with improved prognosis in presence of adjuvant chemotherapy in patients with TNBC in 2009 node-positive BC samples from the BIG 02-98 adjuvant phase III trial [20]. These results are well in line with our findings.

In a pooled analysis of five phase II studies that included patients (*n* = 161) with TNBC treated with neoadjuvant platinum-based chemotherapy, Telli et al. reported that stromal TIL density was associated with RCB 0/1 status, though it was not significant in multivariable analysis when homologous recombination deficiency status was included [21]. The results are consistent with our finding that higher sTIL-0 values are favorably associated with pCR in the ADAPT TN trial.

Besides an increasing body of scientific evidence regarding the prognostic and predictive value of baseline sTIL values in breast cancer, there are yet limited data regarding the association between dynamic sTIL measurements during neoadjuvant chemotherapy and either pCR or IDFS. Skriver et al. analyzed repeated measurements of sTILs among 106 patients with ER positive, HER2 negative, operable breast cancer concerning response to neoadjuvant letrozole therapy. They observed an increase in mean sTIL concentration of about 7% during treatment as well as an association between an increase in sTILs and *lower* probability of pathological response. In fact, the authors suggested that this might provide a rationale for introducing immunotherapy to patients with limited response to endocrine therapy [22]. In our TNBC trial, there was no evidence for association of sTIL increases with pCR or iDFS. However, the situations in different breast cancer subtypes with differing therapies are hardly comparable, not to mention that only one patient had pCR. In a similar analysis among 104 cases of patients with TNBC, changes in sTILs were reported to be associated with improved disease-free survival (DFS) compared to unchanged levels [23]

### Limitations and lessons learned

There are some limitations and lessons learned: Aside from the limited follow-up of three years, the main complications in the current analysis relate to sTIL measurements at week three: The sTIL-3 level was not quantifiable in a significant proportion of patients, often due to low cellularity. Recognizing that low-cellularity status represents a biological feature of response to therapy (characterized by tumor necrosis resulting from extensive response to neoadjuvant therapy), we included this feature as a separately coded category and found that it is indeed informative at least for pCR, though not for iDFS. The possibility of limited tissue due to extensive NACT response should be taken into account explicitly in designing future trials looking at early response assessment in any breast cancer subtype (lessons learned). Furthermore, upon interpretation of our results one has to consider that neither chemotherapy duration of 12 weeks nor anthracycline-free chemotherapy are yet considered standard in patients with TNBC, who are still considered to have high-risk disease. Finally, it has repeatedly been reported that lack of adequate standardization and training render visual sTILs assessment subject to inter- and intraobserver variability [16, 24, 25]. We performed our analyses according to the “TIL Working Group,” which has aimed to standardize sTIL assessment in tumors including breast cancer [26] with the goal of facilitating reproducibility and clinical adoption. In fact, independent observer concordance rates were excellent, particularly at a cutoff of 30%. Nevertheless, modern analytical methods such as computational sTIL assessment might further improve TIL analyses in the future [27, 28].

In general, triple-negative disease is characterized by an unfavorable prognosis [29]; however, prognosis is more favorable among patients with excellent response to neoadjuvant chemotherapy [3, 4]. For patients likely to have pCR, de-escalation of (neoadjuvant) chemotherapy to alleviate treatment toxicity has become an option [8]. In a previous analysis of ADAPT-TN, we demonstrated that proliferation biomarkers such as PAM50 proliferation, ROR scores (all *p* < .004), and higher Ki-67 (IHC; *p* < .001) were positively associated with pCR. In the nab-paclitaxel/carboplatin study arm, expression of immunological (CD8, PD1, and PDL1) genes and proliferation markers (proliferation and ROR scores, MKI67, CDC20, NUF2, KIF2C, CENPF, EMP3, and TYMS) were positively associated with pCR (*p* < .05 for all). To the extent that associations of markers with pCR translate into associations with survival, these markers as well as sTILs studied here could be used to more accurately characterize risk groups and ultimately to construct selection criteria to define candidates for chemotherapy de-escalation in the future [30]. However, simplistic criteria based solely on pCR could be misleading: In the current trial, high immune response at 3 weeks was favorable for pCR and independently for iDFS; pCR is a marker for iDFS but not necessarily a mediator, suggesting that immune response may support eradication of both primary and metastatic clones. On the other hand, even though neoadjuvant NP/C was more effective than NP/G at destroying primary tumor (evidenced by higher rates of low cellularity and pCR), we did not see evidence that this superiority will translate into a long-term survival advantage.

## Conclusion

The current analysis provides strong evidence that immune processes marked by higher sTIL measurements (either at baseline or after 3 weeks) are associated not only with favorable response to neoadjuvant chemotherapy, but also with favorable survival. In line with recently published results on excellent survival in young TNBC patients with high TILs [31] and no systemic treatment, our analysis strongly motivates investigation of tailored (deescalated) treatment concepts in early-TNBC patients with favorable biomarker profile (including sTILs) in future prospective trials, with the aim of optimal individualized care for every patient. The “added value” of sTILs for iDFS (i.e., their independent prognostic impact beyond pCR) could favor de-escalation trial designs including iDFS as a primary or at least co-primary endpoint in TNBC.

## Supplementary Information

**Additional file 1. Figure S1.** Consort diagram.

**Additional file 2. Figure S2.** Distribution of semi quantitative measurements of A sTIL-0 and B sTIL-3 (*n* = 336).

**Additional file 3. Figure S3.** ROC curves and AUC in all patients regarding. A sTIL-0 and B sTIL-3

**Additional file 4. Table S1.** Mediation analysis for raw sTIL-0 and sTIL-3 measurements as continuous variables. **Table S2.** Mediation analysis for categorized sTIL-0 and sTIL-3 measurements.

## Data Availability

The datasets used and/or analyzed during the current study are available from the corresponding author on reasonable request.

## Abbreviations

AUC: Area under the ROC curve
CD8: Cluster of differentiation 8
CD20: Cluster of differentiation 20
CENPF: Centromere protein F
CI: Confidence interval
cN: Clinical nodal status
cT: Clinical tumor status
DDFS: Distant disease-free survival
DFS: Disease-free survival
EMP3: Epithelial membrane protein 3
ER: Estrogen receptor
HER2: Human epithelial receptor 2
HR: Hazard ratio
IDFS: Invasive disease-free survival
KIF2c: Kinesin-like protein 2C
NP/C: Nab-paclitaxel/carboplatin chemotherapy
NP/G: Nab-paclitaxel/gemcitabine
NUF2: Kintochore protein Nuf 2
OS: Overall survival
pCR: Pathological complete response
PD1: Programmed death receptor 1
PDL1: Programmed death receptor ligand 1
PR: Progesterone receptor
RD: Residual disease
ROR: Risk of recurrence
SD: Standard deviation
sTIL-0: Stromal tumor-infiltrating lymphocytes at baseline
sTIL-3: Stromal tumor-infiltrating lymphocytes at 3 weeks
sTILs: Stromal tumor-infiltrating lymphocytes
TNBC: Triple-negative breast cancer
TYMS: Thymidylate synthase
Yrs: Years
