# Supplementary figures and images for: Impact of stromal tumor-infiltrating lymphocytes (sTILs) on response to neoadjuvant chemotherapy in triple-negative early breast cancer in the WSG-ADAPT TN trial
Source: Breast Cancer Res. 2022 Sep 2;24:58. doi: 10.1186/s13058-022-01552-w (PMC9438265; doi:10.1186/s13058-022-01552-w)

Suppl Figure 1

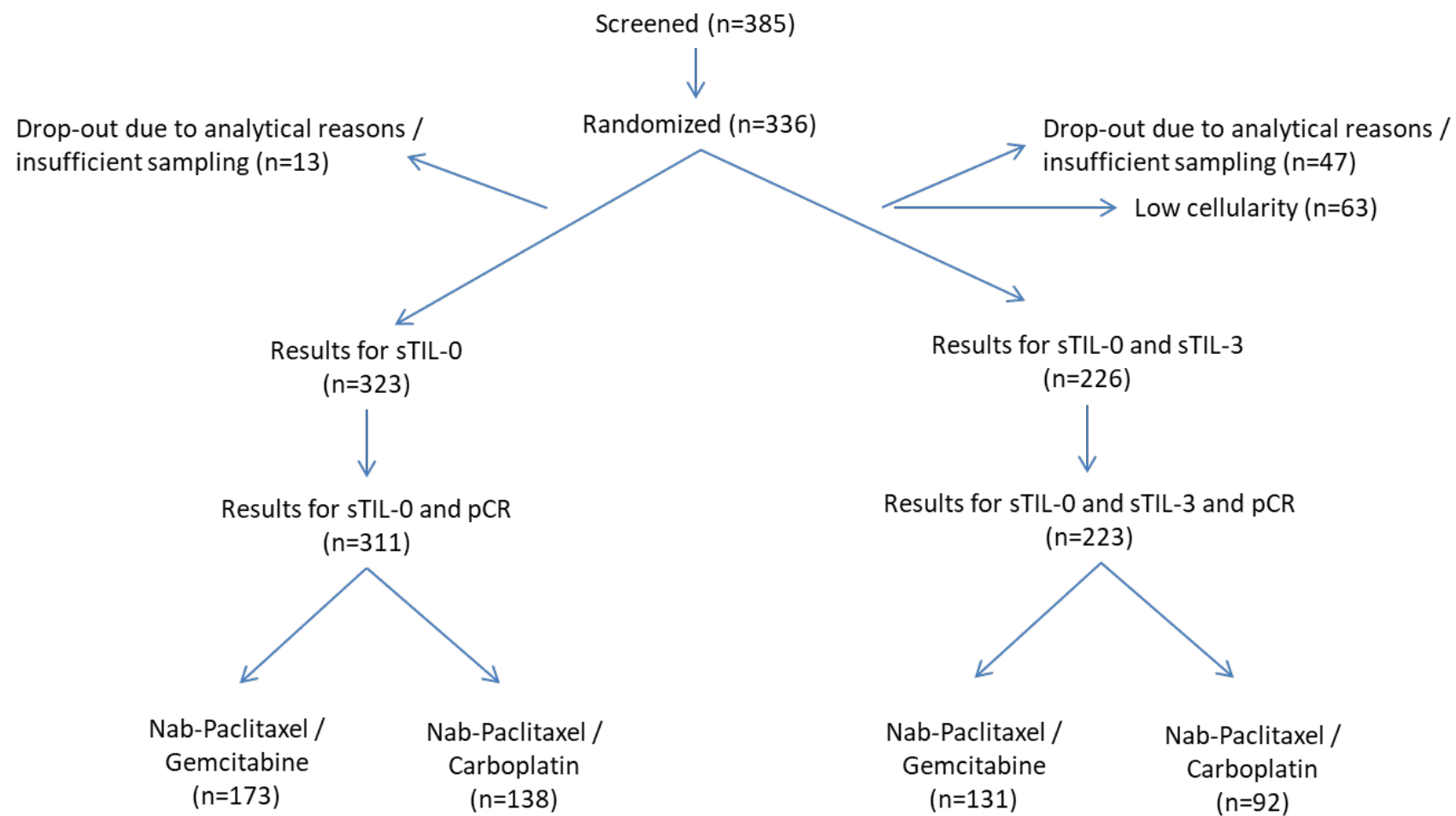

Supplement: Supplementary file 1 — Additional file 1. Figure S1. Consort diagram. [file 13058_2022_1552_MOESM1_ESM.pdf]

Suppl Figure 2

3A

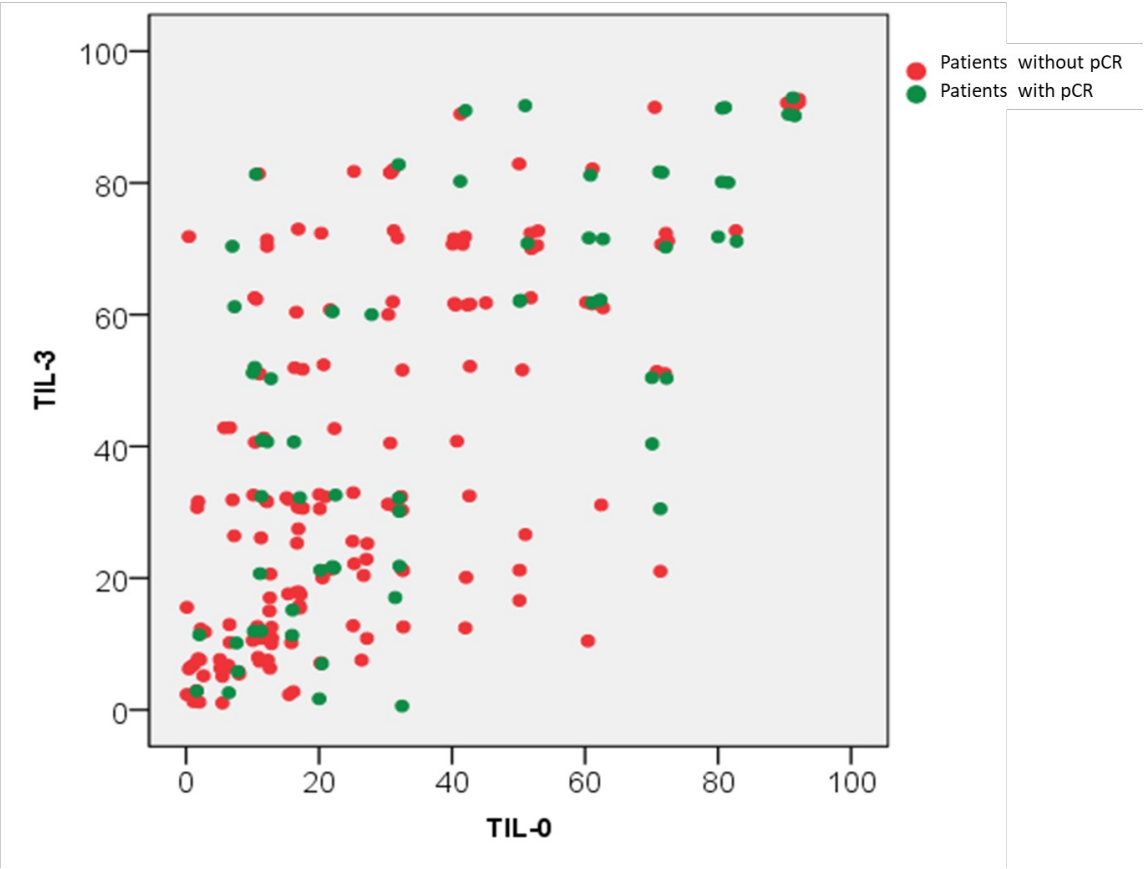

Supplement: Supplementary file 2 — Additional file 2. Figure S2. Distribution of semi quantitative measurements of A sTIL-0 and B sTIL-3 (n = 336). [file 13058_2022_1552_MOESM2_ESM.pdf]

Suppl Figure 3

A

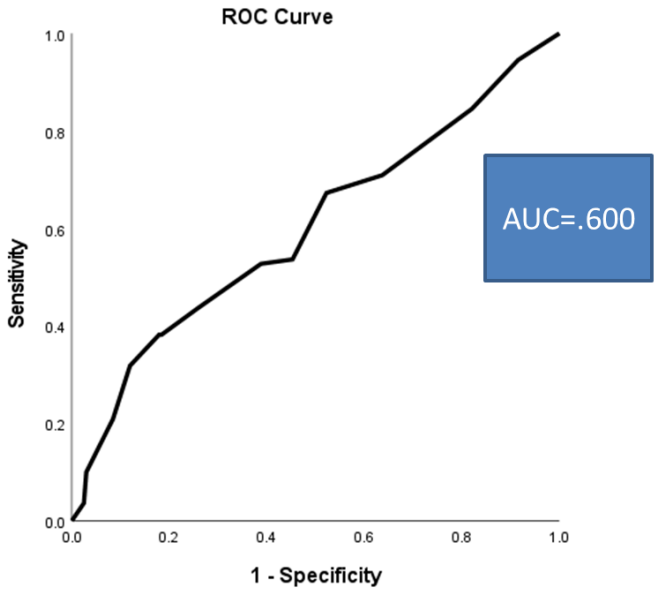

B

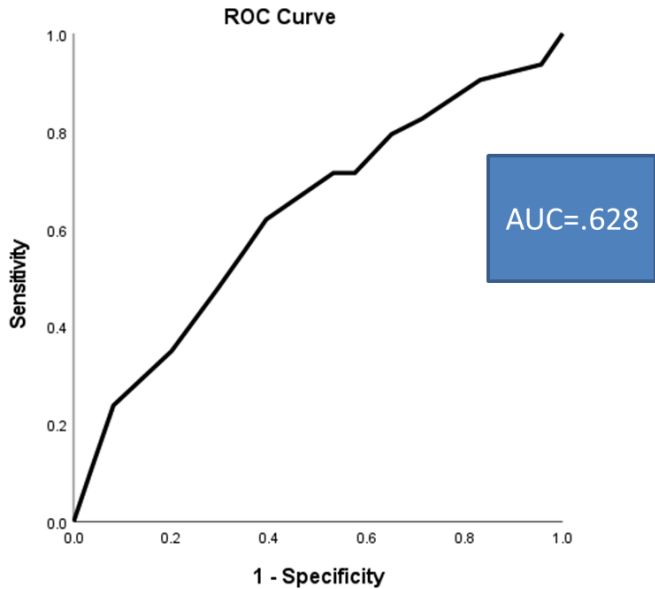

Supplement: Supplementary file 3 — Additional file 3. Figure S3. ROC curves and AUC in all patients regarding. A sTIL-0 and B sTIL-3 [file 13058_2022_1552_MOESM3_ESM.pdf]
