# Supplementary material for: Impact of stromal tumor-infiltrating lymphocytes (sTILs) on response to neoadjuvant chemotherapy in triple-negative early breast cancer in the WSG-ADAPT TN trial
Source: Breast Cancer Res. 2022 Sep 2;24:58. doi: 10.1186/s13058-022-01552-w (PMC9438265; doi:10.1186/s13058-022-01552-w)
Supplement: Supplementary file 4 — Additional file 4. Table S1. Mediation analysis for raw sTIL-0 and sTIL-3 measurements as continuous variables. Table S2. Mediation analysis for categorized sTIL-0 and sTIL-3 measurements. [file 13058_2022_1552_MOESM4_ESM.docx]

**Suppl. Table 1:** Mediation analysis for raw sTIL-0 and sTIL-3 measurements as continuous variables.

| **Mediation analysis / factors** | **p** | **HR** | **Lower** | **Upper** |
| --- | --- | --- | --- | --- |
| **univariate model (sTIL-0 --> iDFS)** |  |  |  |  |
| sTIL-0 per 10% increase | **.019** | **.871** | **.777** | **.977** |
| **Multivariate model (sTIL-0 --> pCR --> iDFS)** |  |  |  |  |
| pCR | <.001 | .246 | .117 | .520 |
| sTIL-0 per 10% increase | .104 | .908 | .808 | 1.020 |
|  |  |  |  |  |
| **univariate model (sTIL-3 --> iDFS)** |  |  |  |  |
| sTIL-3 per 10% increase | **.004** | **.845** | **.754** | **.946** |
| **Multivariable model (sTIL-3 --> pCR --> iDFS)** |  |  |  |  |
| pCR | **.005** | **.226** | **.081** | **.633** |
| sTIL-3 per 10% increase | **.022** | **.874** | **.779** | **.981** |
|  |  |  |  |  |

**Suppl Table 2:** Mediation analysis for categorized sTIL-0 and sTIL-3 measurements.

| **Mediation analysis / factors** | **p** | **HR*** | **Lower** | **Upper** |
| --- | --- | --- | --- | --- |
| **univariate model (sTIL-0 --> iDFS)** |  |  |  |  |
| TIL+ vs. TIL- | 0.121 | 0.556 | 0.265 | 1.168 |
| **Multivariate model (sTIL-0 --> pCR --> iDFS)** |  |  |  |  |
| pCR | **<.001** | **0.237** | **0.112** | **0.502** |
| TIL+ vs. TIL- | 0.498 | 0.771 | 0.364 | 1.635 |
|  |  |  |  |  |
| **univariate model (sTIL-3 --> iDFS)** |  |  |  |  |
| 3wTIL+ vs.3wTIL- | **0.018** | **0.416** | **0.202** | **0.858** |
| 3wLC vs. 3wTIL- | 0.133 | 0.600 | 0.308 | 1.169 |
| **Multivariable model (sTIL-3 --> pCR --> iDFS)** |  |  |  |  |
| pCR | **0.001** | **0.282** | **0.135** | **0.590** |
| 3wTIL+ vs. 3wTIL- | **0.048** | **0.481** | **0.233** | **0.993** |
| 3wLC vs. 3wTIL- | 0.890 | 0.952 | 0.475 | 1.910 |
|  |  |  |  |  |
